# Machine Learning-Based Prediction of the Outcomes of Cochlear Implantation in Patients With Cochlear Nerve Deficiency and Normal Cochlea: A 2-Year Follow-Up of 70 Children

**DOI:** 10.3389/fnins.2022.895560

**Published:** 2022-06-23

**Authors:** Simeng Lu, Jin Xie, Xingmei Wei, Ying Kong, Biao Chen, Jingyuan Chen, Lifang Zhang, Mengge Yang, Shujin Xue, Ying Shi, Sha Liu, Tianqiu Xu, Ruijuan Dong, Xueqing Chen, Yongxin Li, Haihui Wang

**Affiliations:** ^1^Key Laboratory of Otolaryngology Head and Neck Surgery, Department of Otorhinolaryngology Head and Neck Surgery, Beijing Tongren Hospital, Ministry of Education, Capital Medical University, Beijing, China; ^2^Laboratory of Haihui Data Analysis, School of Mathematical Sciences, Beihang University, Beijing, China; ^3^Beijing Tongren Hospital, Beijing Institute of Otolaryngology, Capital Medical University, Beijing, China

**Keywords:** cochlear nerve deficiency, cochlear implantation, machine learning, stability selection, support vector machines

## Abstract

Cochlear nerve deficiency (CND) is often associated with variable outcomes of cochlear implantation (CI). We assessed previous investigations aiming to identify the main factors that determine CI outcomes, which would enable us to develop predictive models. Seventy patients with CND and normal cochlea who underwent CI surgery were retrospectively examined. First, using a data-driven approach, we collected demographic information, radiographic measurements, audiological findings, and audition and speech assessments. Next, CI outcomes were evaluated based on the scores obtained after 2 years of CI from the Categories of Auditory Performance index, Speech Intelligibility Rating, Infant/Toddler Meaningful Auditory Integration Scale or Meaningful Auditory Integration Scale, and Meaningful Use of Speech Scale. Then, we measured and averaged the audiological and radiographic characteristics of the patients to form feature vectors, adopting a multivariate feature selection method, called stability selection, to select the features that were consistent within a certain range of model parameters. Stability selection analysis identified two out of six characteristics, namely the vestibulocochlear nerve (VCN) area and the number of nerve bundles, which played an important role in predicting the hearing and speech rehabilitation results of CND patients. Finally, we used a parameter-optimized support vector machine (SVM) as a classifier to study the postoperative hearing and speech rehabilitation of the patients. For hearing rehabilitation, the accuracy rate was 71% for both the SVM classification and the area under the curve (AUC), whereas for speech rehabilitation, the accuracy rate for SVM classification and AUC was 93% and 94%, respectively. Our results identified that a greater number of nerve bundles and a larger VCN area were associated with better CI outcomes. The number of nerve bundles and VCN area can predict CI outcomes in patients with CND. These findings can help surgeons in selecting the side for CI and provide reasonable expectations for the outcomes of CI surgery.

## Introduction

Cochlear nerve deficiency (CND) is defined as a small or absent cochlear nerve (CN) (Adunka et al., 2007). When the CN is small, it is referred to as cochlear nerve hypoplasia (CNH). When the CN is absent, it is referred to as cochlear nerve aplasia (CNA). The estimated prevalence of CND is 18% among children with congenital sensorineural hearing loss (SNHL) (Jallu et al., 2015).

Cochlear implant (CI) was an effective treatment to restore hearing for patients with SNHL. The mechanism of cochlear implantation (CI) involves converting acoustic signals into electrical signals, directly stimulating the spiral ganglion neurons (SGNs), and transmitting the signals through the CN fibers to the auditory brainstem. Recent years, optics has been proposed to stimulate CN such as optical wireless CI and all-optical CI (Trevlakis et al., 2019, 2020). These architectures could convert acoustic to optical signals which improved the reliability and the efficiency of the transcutaneous link (Boulogeorgos et al., 2021).

In CND patients, due to the decrease in the absolute number of CN fibers, SGNs are insufficient and the stimuli that can be received are limited. In early studies, CND was considered a contraindication for CI (Shelton et al., 1989). However, a large number of studies have shown that some patients with CND can benefit from CI (Kang et al., 2010; Wu et al., 2015; Wei et al., 2017). When compared with patients without CND, patients with CND need higher stimulation to induce a CN response (Yousef et al., 2021). Generally, children with CND perform worse than those without CND (Wei et al., 2017; Yousef et al., 2021) and some patients even experience no benefit at all (Colletti et al., 2004). Due to the low incidence of CND and the uncertainty regarding the effects of CI surgery, there has been no study involving a large sample of patients with CND. At the same time, patients with CND were far more likely to exhibit inner ear malformations than patients without CND (Wu et al., 2015), which affected the number of SGNs and further limited the CI outcomes, making it difficult to determine whether the surgical results were different due to the differences in the surgical methods and electrode positions (Shi et al., 2019). Some studies have revealed the predictive role of radiographic information for CI outcomes in CND patients (Wu et al., 2015), but they did not include the patients with inner ear malformations.

Over the past 5 years, machine learning has been increasingly used to automate intelligent processes and improve the efficiency of medical processes. For example, cochlear implants can be enhanced by adopting machine learning techniques, which have been applied to create predictive models (Crowson et al., 2020; Velde et al., 2021) (see Velde et al., 2021 for a recent review). In addition, machine learning algorithms have been used to predict cochlear implantation (CI) outcomes. The prediction of postoperative CI performance from preoperative data may allow practitioners to evaluate implantation candidacy, estimate performance expectations from non-modifiable predictors, and optimize the procedure by intervening in modifiable predictors (Crowson et al., 2020). Han et al. (2019) established a multiple regression model for 25 CND patients with normal cochlea to predict CI postoperative outcomes, explaining 66% variance of the Categories of Auditory Performance (CAP) scores for patients with 2-year CIs. They concluded that the postoperative effect of CI in patients with CND was related to the preoperative auditory brainstem response (ABR) and the area ratio of the vestibulocochlear nerve (VCN) to the facial nerve (FN). Preoperative counseling based on this model helped determine the treatment modalities for hearing rehabilitation.

In this study, we analyzed the CI surgery-related factors in CND patients with normal cochlea, based on a relatively larger sample. We used a data-driven multivariate approach based on machine learning to evaluate postoperative hearing and speech rehabilitation in patients with CND and the influencing factors. We retrospectively analyzed the clinical data of CI surgery from 70 patients with CND and normal cochlea. Then, we measured and averaged audiological and radiological features of patients with CND to form feature vectors. Data-driven methods (i.e., stability selection and SVM) were applied to data from patients with CND to relate various factors to the CI outcomes and to build the corresponding predictive models. In addition, stability selection, a machine learning method that identifies highly consistent and representative features, was used to examine the factors that best differentiate the effects of postoperative hearing and speech rehabilitation in patients with CND.

## Materials and Methods

### Participants

This study was approved by the Research Ethics Board of Tongren Hospital, Beijing, China. We considered 70 CI pediatric recipients (37 males and 33 females; ages 7–54 months) with CND who were diagnosed using three-dimensional MRI and who underwent CI between January 2012 and August 2018 in this study. All children failed to pass the newborn hearing screening sequence and the ear with better residual hearing was selected to undergo CI. Thirty-four patients were implanted with Med-El (Innsbruck, Austria) devices, 22 with Cochlear (Sydney, Australia) devices, 14 with AB (California, United States) devices. Table 1 lists the demographic details with quantitative variables shown as count, mean, standard deviation, minimum, maximum, and quantile, and qualitative variables shown as count. Hearing impairment was classified according to the World Health Organization classification (Olusanya et al., 2019) into mild (26–40 dB), moderate (41–60 dB), severe (61–80 dB), and profound (81 dB or greater). The inclusion criteria were as follows: (1) the diameter of the CN smaller than that of the FN or less than four nerve bundles within the internal auditory canal (IAC), (2) bilateral severe to profound SNHL, (3) no inner ear malformation or other congenital syndromes, (4) history of CI, and (5) completion of 2-year follow-up after CI.

**TABLE 1:** Descriptive statistics of patients.

	Count	Mean	Standard deviation	Min.	25%	50%	75%	Max.
Age (months)	70.00	27.31	13.92	7.00	14.00	25.50	38.00	54.00
Residual hearing (dB)	70.00	108.17	14.07	81.00	97.50	106.88	124.69	125.00
Bony cochlear nerve canal diameter (mm)	70.00	0.83	0.58	0.01	0.35	0.83	1.17	2.28
Internal auditory canal diameter (mm)	70.00	2.47	0.85	0.41	1.91	2.51	2.96	4.42
Number of nerve bundles	70.00	1.71	0.80	1.00	1.00	2.00	2.00	4.00
Vestibulocochlear nerve area (mm^2^)	70.00	1.32	0.55	0.30	0.91	1.27	1.71	2.78
Area ratio of the vestibulocochlear nerve to the facial nerve	70.00	1.50	0.48	0.44	1.22	1.47	1.69	2.63
40-Hz auditory-evoked related potential	70.00	–	–	–	–	–	–	–
Auditory brainstem responses	70.00	–	–	–	–	–	–	–
Cochlear microphonics	70.00	–	–	–	–	–	–	–
Distortion product otoacoustic emissions	70.00	–	–	–	–	–	–	–
Acoustic immittance	70.00	–	–	–	–	–	–	–
CAP	70.00	4.10	1.32	2.00	3.00	5.00	5.00	7.00
SIR	70.00	1.87	0.92	1.00	1.00	2.00	3.00	4.00
MAIS	70.00	25.14	10.47	3.00	18.00	26.50	33.75	40.00
MUSS	70.00	11.96	10.32	0.00	3.00	8.00	21.75	33.00

*Not available values (–) indicate discrete variables without mean, standard deviation, maximum, minimum, and quantile.*

### Radiographic Examinations

**FIGURE 1:**
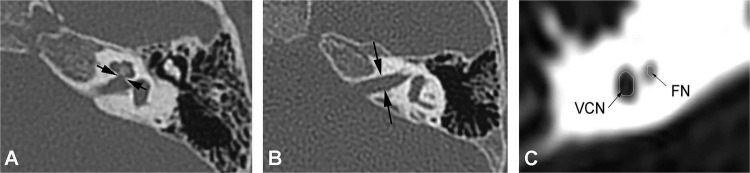
**(A)** Measurement of the bony cochlear nerve canal diameter using high-resolution computed tomography (distance between the two black arrows). **(B)** Measurement of the internal auditory canal diameter using high-resolution computed tomography (distance between the two black arrows). **(C)** Measurement of the area ratio of the vestibulocochlear nerve to the facial nerve at the cerebellopontine angle using magnetic resonance imaging (black arrows).

**FIGURE 2:**
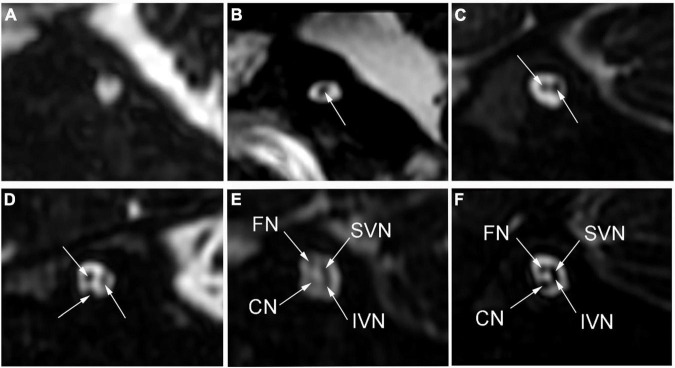
The number of nerve bundles (indicated by the white arrows) in the internal auditory canal on oblique sagittal magnetic resonance imaging. **(A)** No nerve bundle, **(B)** one nerve bundle, **(C)** two nerve bundles, **(D)** three nerve bundles, **(E)** four nerve bundles (thin), **(F)** four nerve bundles (normal); CN: cochlear nerve, FN: facial nerve, IVN: inferior vestibular nerve, SVN: superior vestibular nerve.

High-resolution computed tomography was used to evaluate inner ear malformations according to Sennaroglu’s classification (Sennaroğlu and Bajin, 2017). Normal cochlea was diagnose when normal cochlear appearance shown with current MRI and CT. The diameter of the bony cochlear nerve canal (BCNC) with the width of the canal at the midportion of the IAC fundus (Figure 1A) and the widest diameter of the IAC (Figure 1B) were measured on computed tomography images. The CN traverses along the fundus of the IAC to the base of the modiolus through the BCNC. Magnetic resonance imaging (MRI) was performed to determine the condition of the CN using a 1.5 Tesla scanner or a 3.0 Tesla scanner. The scan sequence is a 3D FIESTA water imaging sequence. Oblique sagittal reconstruction was performed perpendicular to the plane of the IAC. The areas of the VCN and FN at the cerebellopontine angle (CPA) were evaluated, as the cross-sections of these nerves were well visualized as this level. The area ratio of the VCN to the FN was evaluated at the CPA using MRI (Figure 1C). In addition, the number of nerve bundles was counted within the IAC (Figure 2).

### Preoperative Auditory Evaluation

The diagnostic protocol for children with suspected hearing loss incorporated behavioral testing, acoustic emittance, distortion product otoacoustic emission (DPOAE), ABR, cochlear microphonics (CM), and 40-Hz auditory-evoked related potential (40-Hz AERP). The average hearing threshold was assumed to be 5 dB HL greater than the maximum output of the audiometer and was averaged across 0.5, 1.0, 2.0, and 4.0 kHz of pure-tone or behavioral testing.

### Cochlear Implantation Device and Activation

The CI device was selected by the parents with the support and counseling by the CI team. Typically, the first mapping was initiated at 3–4 weeks after the surgery. During the programming sessions, observation and conditioned behavioral audiometry techniques were used to determine the electrical threshold and comfortable listening levels. Usually, a stable map can be achieved at 3–6 months after the initial stimulation.

### Evaluation of Cochlear Implantation Outcomes

**FIGURE 3:**
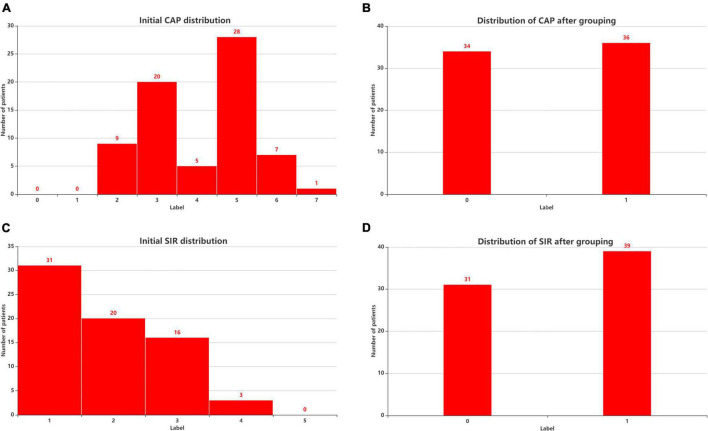
Patient distribution before and after considering CAP and SIR grouping. **(A)** Initial CAP distribution. **(B)** Distribution of CAP after grouping. **(C)** Initial SIR distribution. **(D)** Distribution of SIR after grouping. CAP, categories of auditory performance; SIR, speech intelligibility rating.

Postoperative speech evaluation was performed at 3, 6, 12, 18, and 24 months after CI. Since most of the patients had stable outcomes at 2 years, we selected the 2-year outcomes as the predicted results. The CAP, Speech Intelligibility Rating (SIR), Infant-Toddler Meaningful Auditory Integration Scale (IT-MAIS, for patients aged < 3 years) or Meaningful Auditory Integration Scale (MAIS, for patients aged > 3 years), and Meaningful Use of Speech Scale (MUSS) were used to evaluate hearing and speech in the patients 24 months after CI surgery. The CAP has eight levels of sound perception (0–7), ranging *from no awareness of the environment* (0) to *use of telephone with known users* (7). The CAP is intended to reflect the real-life auditory capabilities of children. The SIR is a highly reliable and time-effective measure of children’s speech production in real-life situations and ranks children’s spontaneous speech into five categories, ranging from connected speech is unintelligible (1) to connected speech is intelligible to all listeners (5). To distinguish the degree of patients’ auditory performance and speech perception, we divided the patients into two groups according to CAP and SIR. Figure 1 shows the initial distributions of CAP (Figure 3A) and SIR (Figure 3C) and the distributions of CAP (Figure 3B) and SIR (Figure 3D) after grouping. For CAP, the patients were divided into spoken language understanding (CAP of 5–7) and no spoken language understanding (CAP of 0–4). For SIR, the patients were divided into intelligible speech (SIR of 2–5) and unintelligible speech (SIR of 1).

### Feature Selection

Feature selection is essential in feature engineering as it aims to find an optimal subset of features and eliminate redundant features for classification. The effectiveness of hearing and speech rehabilitation after receiving a CI depends on various complex and interdependent factors. By considering the experience of doctors, we collected and measured these influencing factors. We measured the radiological and audiological characteristics of patients with CND multiple times and averaged the results. Radiological features include the bony cochlear nerve canal diameter, internal auditory canal diameter, number of nerve bundles, VCN area and area ratio of the VCN to the FN. Audiological features include residual hearing, 40-Hz AERP, ABR, CM, DPOAE, and acoustic immittance. In addition to radiological and audiological features, we considered the implantation age of patients with CND. To build a simple model, we applied feature selection to these 12 features. For feature selection, the variance threshold and stability selection are important methods. In brief, the variance threshold removes all features whose variance does not meet a certain threshold. By default, it removes all zero-variance features, that is, features with the same value across samples.^1^ In this study, we performed preliminary feature selection using the variance threshold, graphical method and correlation method and then applied stability selection to the remaining features. These features were used as inputs to an SVM classifier and stability selection coupled with SVM. As usual for classifiers, we applied min-max normalization to the data before classification and stability selection to ensure that all features had a common scale and range.

### Stability Selection

A robust model should be sufficiently complete to allow generalization and interpretation. Hence, the most salient discriminating features consistent across a range of model parameters should be selected. Stability selection achieves state-of-the-art feature selection while preventing overfitting and enabling data interpretability. In general, representative features do not score 0 for similar features or associative features. We used randomized logistic regression and the randomized least absolute shrinkage and selection operator (LASSO) for stability selection. Labels CAP and SIR are discrete variables. Labels MAIS and MUSS are continuous variables. Therefore, the remaining 6 features and the label CAP or SIR were entered into the randomized logistic regression model; the remaining 6 features and the label MAIS or MUSS were entered into the randomized LASSO model. The stability score of the features to the labels was obtained, and the feature selection was made according to the stability score.

Stability selection used a randomized logistic regression algorithm, which worked by subsampling the training data and fitting a L1-penalized logistic regression model. By performing this double randomization several times (running logistic regression algorithms on different subsets of data and features), the method assigned high scores to features that were repeatedly selected across randomizations. In short, the features selected more often were considered as representative features (Meinshausen and Bühlmann, 2010). Stability selection used a randomized LASSO algorithm, which worked by subsampling the training data and computing a Lasso estimate (Meinshausen and Bühlmann, 2010). In stability selection, the feature stability increases as a feature is increasingly selected over repeated subsampling processes (Nogueira et al., 2017). As stability selection includes internal randomization over many interactions, it yields a more reliable and consistent feature set than conventional filtering or other multivariate approaches (Mahmud et al., 2020).

We considered the regularization parameter C of 1, the scaling parameter of 0.5, a sample fraction of 0.75 and 200 resampling processes to implement randomized logistic regression. The scaling parameter was used to randomly scale the features (Meinshausen and Bühlmann, 2010). We considered regularization parameter alpha = “aic,” sample fraction = 0.75, scaling = 0.5, number of resamples = 200 in our implementation of randomized LASSO. This was not the alpha parameter in the stability selection article which was scaling (Meinshausen and Bühlmann, 2010). Randomized LASSO was able to select the optimal alpha based on “AIC.” The feature scores were scaled between 0 and 1, where 0 was the lowest score (i.e., irrelevant feature) and 1 was the highest score (i.e., most representative or stable feature). Over 200 resampling processes, stability selection provided the overall feature scores (0–1) based on the selection frequency, and a variable was selected. The stability scores were ranked to identify the most important, consistent, stable, and invariant features (i.e., demographic, audiological, and radiological features) over a range of model parameters. We used the ranked features and corresponding class labels in an SVM classifier. Based on the input stable features, the SVM classified patients with CND for different stability thresholds.

### Support Vector Machine Classification

Data-driven multivariate analysis is widely used for modeling complex data and understanding relations between the considered variables. Parameter-optimized SVM classifiers can provide robust discriminative models with small sample sizes, being suitable for human neuroimaging studies (Tan et al., 2015; Feng et al., 2018; Skidmore et al., 2020). The classification performance is greatly affected by the choice of kernel functions, which can map non-linearly separable data onto a linearly separable space. Other tunable parameters, such as the kernel, regularization coefficient C, and γ (γ is an argument having the RBF function as the kernel), also determine the performance. Thus, we used grid search to find the optimal kernel, C, and γ. For kernel functions, we considered the linear function and radial basis function, whereas for C, we considered values from 1 to 10 in increments of 1, and for γ, we considered values of 0.01, 0.02, 0.03, 0.04, 0.05, 0.1, 0.2, 0.3, 0.4, and 0.5. Both C and γ were evaluated using a radial basis function kernel. We randomly split the data into training and test sets containing 80% and 20% of the available samples, respectively.

During training, we fine-tuned parameters C and γ to find the values that maximally distinguish observations from the CI postoperative CAP and SIR in good and poor recovery groups. The SVM learned the support vectors from the training data containing the attributes (e.g., age in months, residual hearing) and class labels (e.g., spoken language understanding). The resulting hyperplanes were fixed with maximum margin of separation between classes and used to predict unseen test data by providing the unlabeled attributes to the model. The classification performance was evaluated using common measures: accuracy, F1-score, and area under the receiver operating characteristic curve (AUC) (Saito and Rehmsmeier, 2015). The AUC describes the degree to which a model can distinguish between classes. An excellent model has an AUC close to 1, indicating high separability, whereas a poor model has an AUC close to 0, indicating poor separability.

### Technology Roadmap

**FIGURE 4:**
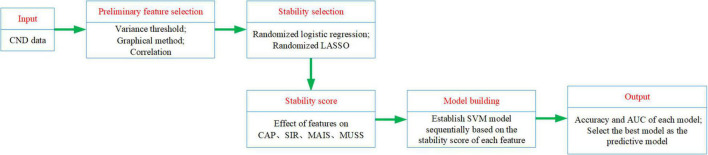
Technology Roadmap.

**FIGURE 5:**
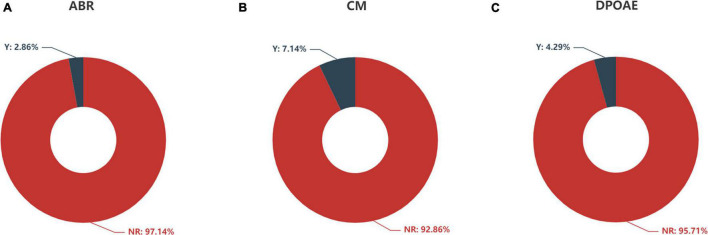
Feature distribution of **(A)** ABR, **(B)** CM, and **(C)** DPOAE according to auditory response. ABR, auditory brainstem response; CM, cochlear microphonics; DPOAE, distortion product otoacoustic emission; Y, response; NR, no response.

**FIGURE 6:**
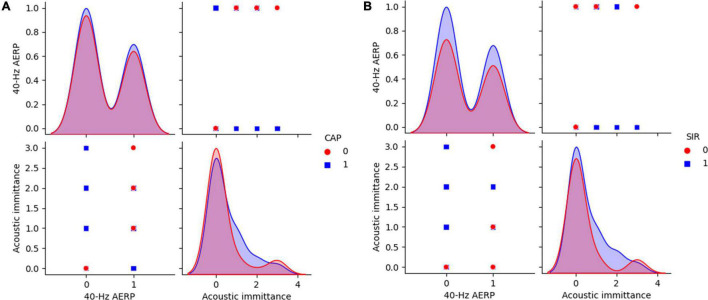
40-Hz AERP and acoustic immittance for CAP and SIR. **(A)** CAP: 0 indicates no spoken comprehension, and 1 indicates spoken comprehension. **(B)** SIR: 0 indicates unintelligible connected speech, and 1 indicates intelligible connected speech. CAP, categories of auditory performance; SIR, speech intelligibility rating.

**FIGURE 7:**
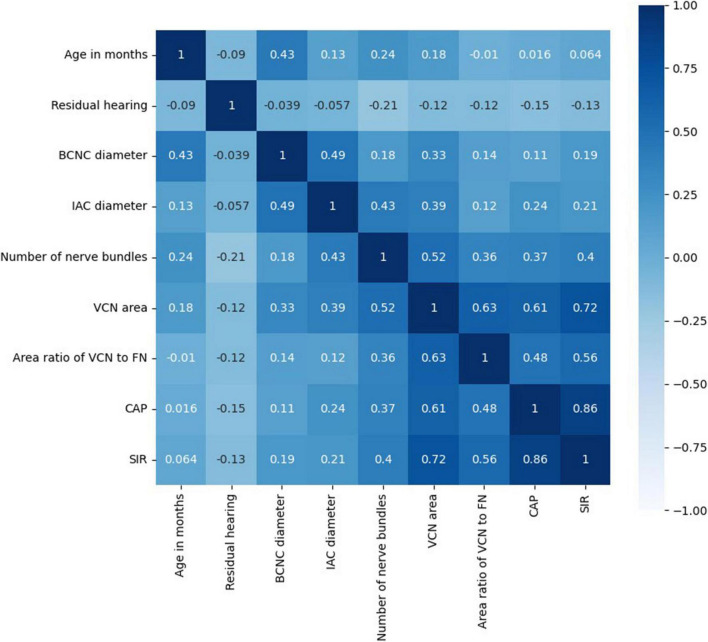
Correlation coefficient matrix.

**FIGURE 8:**
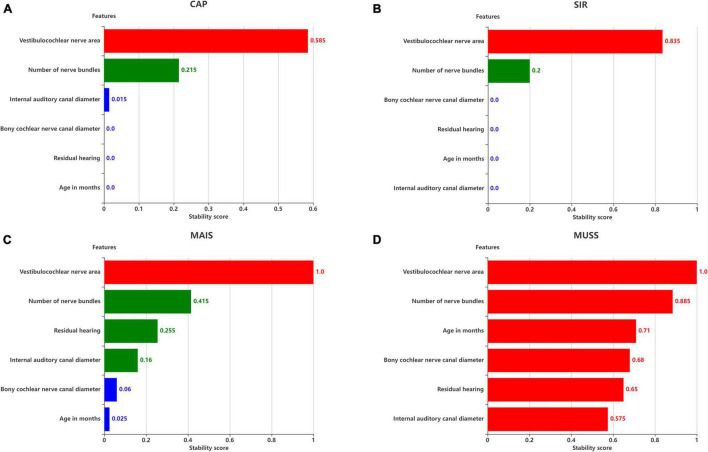
Stability scores for feature selection. Effect of features on **(A)** SIR, **(B)** CAP, **(C)** MAIS, and **(D)** MUSS. The stability score in 0–1 is shown as range per bin.

The experimental process is shown in Figure 4. We input the CND dataset. First we made a preliminary feature selection. As shown in Figure 5, we removed three features according to the variance threshold. As shown in Figure 6, we removed two features according to the effect of the features on the labels, that is, the graphical method. As shown in Figure 7, we removed one feature according to the correlation. Stability selection was made for the remaining six features. We entered 6 features and a label CAP or SIR into a randomized logistic regression model. We entered 6 features and a label MAIS or MUSS into a randomized LASSO model. We got the stability score of the features on the labels and sorted the stability scores as shown in Figure 8. According to the sorted features, the features were added to the SVM model in turn, and the models labeled CAP and SIR were established, respectively. Accuracy and AUC of each model were output, and the best models were selected, respectively, as the prediction models for predicting auditory and speech performance after CI.

## Results

### Feature Selection

The distributions of ABR, CM and DPOAE are shown in Figure 5. For ABR, 68 patients showed no hearing response (NR), and 2 patients had a hearing response (Y), while for CM, 65 patients had NR and 5 had Y, and for DPOAE, 67 patients had NR and 3 had Y. Variance selection removed the ABR, CM, and DPOAE. The influences of the 40-Hz AERP and acoustic immittance on the labels are shown in Figure 6. As these two features had less influence on the SIR and CAP labels, they were removed. The correlation coefficient matrix is shown in Figure 7. Considering that the correlation coefficient between area ratio of the VCN to the FN and VCN area is 0.63, it has a high correlation. Area ratio of the VCN to the FN is removed.

The six remaining features and the corresponding labels were processed using stability selection to obtain the most representative factors affecting the postoperative hearing and speech rehabilitation of patients with CND and CI. Figure 8 illustrates the importance of stability selection. Among the factors affecting the postoperative CAP and SIR in patients with CND, VCN area and number of nerve bundles were highly stable and important. Therefore, these features were selected to establish a prediction model of postoperative CAP and SIR in patients with CND. Among the factors affecting the postoperative MAIS in patients with CND, VCN area was the most stable, and the stability scores of the number of nerve bundles, residual hearing, and internal auditory canal diameter were similar. Among the factors affecting the postoperative MUSS in patients with CND, VCN area was the most stable, with a stability score of 1, followed by the number of nerve bundles. Overall, characteristic VCN area and number of nerve bundles were more stable and showed the greatest influence on the postoperative hearing and speech rehabilitation of patients with CND.

### Support Vector Machine Classification of Hearing and Speech Rehabilitation Effects Using Vestibulocochlear Nerve Area and Number of Nerve Bundles

We only used VCN area and number of nerve bundles to analyze the effects of postoperative hearing and speech rehabilitation in patients with CND. These features and the corresponding category labels were used to train the SVM. In addition, we applied sevenfold cross-validation and grid search during training to determine the optimal SVM parameters. The optimal parameters for the maximum classification performance listed in Table 2 were *C* = 5 and γ = 0.02 for CAP and *C* = 6 and γ = 0.2 for SIR.

**TABLE 2:** Maximum performance (%) of SVM classifier for distinguishing hearing and speech rehabilitation effects (good and poor).

Measure	CAP	SIR
Accuracy	71	93
AUC	71	94
F1-score	67	93

*F1-score = 2 (precision × recall)/(precision + recall).*

We then selected the best model and performance measures from the predicted class labels, which were obtained from the unseen test data and corresponding ground truth. We applied the SVM classifier using VCN area and number of nerve bundles to identify the effects of hearing and speech rehabilitation. Table 2 shows that for the hearing rehabilitation effect considering VCN area and number of nerve bundles, the accuracy of spoken language understanding prediction after CI surgery in patients with CND was 71%. For the speech rehabilitation effect considering those two features, the accuracy of intelligible connected speech prediction after CI surgery in patients with CND was 93%. These results indicate suitable prediction of the effects of postoperative hearing and speech rehabilitation after receiving a CI.

**FIGURE 9:**
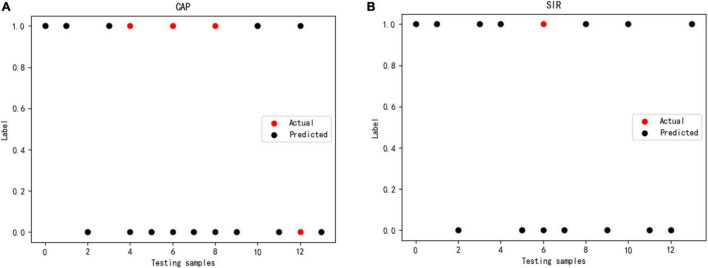
Prediction using SVM classifier for **(A)** CAP and **(B)** SIR.

The SVM classification results on the test dataset are shown in Figure 9. As a correct prediction is shown with a black circle, a model with fewer red circles is preferable, whereas numerous red circles indicate a low generalization ability of the SVM classifier. In fact, each red circle indicates a misclassified patient with CND. Figure 9A shows four red circles, indicating four patients with misclassified CAP and a classification accuracy of 71%. Figure 9B shows one red circle, indicating that the SIR of only one patient was misclassified and a classification accuracy of 93%.

### Stability Selection for Support Vector Machine Training

**FIGURE 10:**
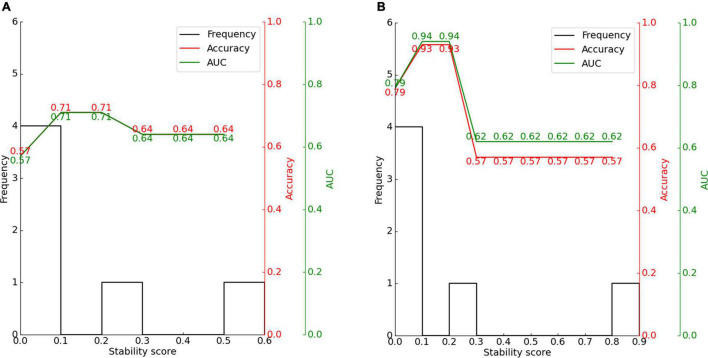
Effect of stability score threshold on model performance for **(A)** CAP and **(B)** SIR. The stability score ranges from 0 to 1.

We then used stability selection to identify the most representative stable features to separate groups without overfitting. We evaluated stability thresholds yielding different classification performances. The effect of the stability selection threshold on the classification performance is shown in Figure 10A for CAP and in Figure 10B for SIR. The histogram shows the distribution of feature scores.

The feature scores for stability selection were first determined. As shown in Figure 8, for CAP, stability in descending order was obtained for VCN area, number of nerve bundles, internal auditory canal diameter, bony cochlear nerve canal diameter, residual hearing, and age in months; for SIR, this order was obtained for the VCN area, number of nerve bundles, bony cochlear nerve canal diameter, residual hearing, age in months, and internal auditory canal diameter.

The features obtained by stability selection were used in the SVM, whose performance depended on the stability threshold. For CAP and SIR, 66.7% of features scored between 0 and 0.1. Hence, most features were selected less than 10% of the time over 200 model iterations and thus carried near-zero importance for separating groups. Therefore, 66.7% of the features were not related to the grouping of CAP or SIR.

For CAP, the maximum classification performance with 71% accuracy, 71% AUC, and F1-score of 67% was achieved for a stability score threshold of 0.10. For this threshold, two out of the six features were selected. For SIR, stability selection provided two out of the six features (33.3%), reaching an accuracy of 93%, AUC of 94%, and F1-score of 93%. Below the optimal threshold of 0.2, the classifier performance reduced owing to the inclusion of unrelated features, while above the threshold, some representative features for distinguishing the effects of hearing and speech rehabilitation were discarded. Even when choosing a stability threshold of 0.5 as a conservative selection, CAP classification reached 64% accuracy with one selected feature, and SIR classification reached 57% accuracy with one selected feature. Thus, predicting the effects of CIs on postoperative hearing and speech rehabilitation in patients with CND may require only a few representative features to notably outperform the random level of classification.

## Discussion

The present study included 70 patients diagnosed with CND with normal cochlea according to computed tomography and MRI findings. All subjects underwent unilateral CI. We summarized the age at operation, preoperative audiological findings, and preoperative imaging characteristics of CND patients with normal cochlea and analyzed their correlation with the 2-year postoperative outcome of CI. In our study, the overall mean scores of the CAP, SIR, IT-MAIS/MAIS, and MUSS after 2-year CI activation were 4.10 ± 1.32, 1.87 ± 0.92, 25.14 ± 10.47 and 11.96 ± 10.32, respectively. Among the 70 participants, 36 (51.4%) achieved an understanding of common phrases or the ability to carry on a conversation without lip-reading (CAP 5–7) and 39 (55.7%) patients achieved intelligible speech (SIR > 1). We also obtained a prediction model of the CAP and SIR scores at 2 years after surgery based on these associated factors. We observed that the CAP and SIR scores at 2 years after CI surgery were strongly correlated with the number of nerve bundles and the VCN area.

In our study, the mean age at CI was 27.31 ± 13.92 months (range: 7–54 months). All children failed to pass the newborn hearing screening sequence. Age at CI is known to potentially influence the CI outcomes (Peng et al., 2017). Cochlear implantation provides a unique opportunity to study cortical plasticity associated with long-term deafness and restoration of the auditory modality (Lee et al., 2006). Children who underwent implantation at a younger age have been reported to demonstrate greater gains in speech perception over time than those who underwent implantation at an older age (Zwolan et al., 2004). In our study, we did not find a strong correlation between age at CI and CI performance. This might be due to the limited amount of CN and auditory stimulation. The results were consistent with those of previous studies (Birman et al., 2016; Han et al., 2019).

The residual hearing threshold is one of the most important prognostic factors correlated with the CI outcomes (Chiossi and Hyppolito, 2017). It represents the number of SGNs and the integrity of neural pathways including the SGNs and the CN. Patients with residual hearing displayed significant improvements in language development (Carlson et al., 2015) after CI surgery. However, we did not find a significant correlation between the average residual hearing threshold and CI performance, which is consistent with the finding in a previous study (Han et al., 2019). This might be attributed to the poor residual hearing of these CND patients. Moreover, we assumed the average hearing threshold of the absence of measurable response in pure-tone audiometry or behavior test to be equal to the maximum output or 5 dB greater than the maximum output of the audiometer for the purpose of evaluation. The calculated mean residual hearing threshold was 108.17 ± 14.07 dB (range: 81–125 dB), which might have reduced the difference between cases with and without residual hearing.

Auditory brainstem response (ABR) represents the efficacy of hearing aids and cortical development with acoustic stimulation before CI. It was significantly correlated with CI performance in a previous study (Han et al., 2019). However, we did not observe any significant correlation between the ABR and CI outcomes. This finding might be due to the fact that only 2 patients (2/70, 2.86%) exhibited an ABR. Therefore, the sample size was too small to obtain reliable results. Three patients (3/70, 4.27%) exhibited the presence of DPOAE and 5 patients (5/70, 7.14%) exhibited the presence of CM with absent ABR, which suggested a gross discrepancy between the measures of cochlear and neural function in the auditory system diagnosed with auditory neuropathy (AN) (Buchman et al., 2006). The responses of DPOAE as well as CM did not show a statistically significant correlation with CI outcomes, probably due to the small sample size.

Due to the limitations of the current imaging techniques, it is difficult to measure the CN parameters directly. Clinically, the IAC diameter, BCNC diameter, area of the VCN, area ratio of the VCN to the FN, and IAC grade can indirectly determine the condition of the CN. These parameters have been reported to predict the effect of CI after surgery (Shelton et al., 1989; Minami et al., 2015; Birman et al., 2016; Wei et al., 2017; Chung et al., 2018; Han et al., 2019). The maximum diameters of the IAC and the BCNC can indirectly reflect the number of CN fibers and are generally considered to be related to CND (Shelton et al., 1989; Chung et al., 2018). In a previous study (Clemmens et al., 2013), BCNC had a sensitivity of 84% and a specificity of 98% for predicting CND, while IAC had a specificity of 98% and a sensitivity of 44%. In our study, the mean IAC diameter was 2.47 ± 0.85 mm and the mean BCNC diameter was 0.83 ± 0.58 mm. The IAC and BCNC diameters exhibited a weak correlation with CI performance.

Cochlear nerve deficiency (CND) diagnosis mainly relies on MRI (Cerini et al., 2006). Measuring the CN parameters on MRI is the most direct way to determine the condition of the CN. However, due to the limited resolution of the currently used MRI devices, the CN is not clearly visualized. In some cases, although CN fibers are present, they are not reflected in the data regarding CN diameter measurements on MRI and the CN cannot even be visualized. VCN contains all the CN fibers. Hence, some scholars defined CND as VCN deficiency or the absence or thin branches of the VCN (Yamazaki et al., 2015). Measuring the VCN diameter at the CPA indirectly reflects the number of CN fibers. In our study, the VCN area showed a strong correlation with CI performance.

Since direct measurement of the VCN diameter is difficult, some scholars have opted to measure the area ratio of the VCN to the FN. The size of the VCN is generally 1.5–2 times the size of the FN and the size of the CN is similar to that of the FN (Giesemann et al., 2012). Minami et al. (Minami et al., 2015) reported the relationship between the relative sizes of the VCN and FN after CI. They observed that 83% of the patients with IAC stenosis had an FN larger than the VCN. Patients whose FN was larger than the VCN had an average score of 1.1 for auditory behavior after CI, while patients whose FN was smaller than the VCN exhibited an average CAP score of 4.1 after CI. Han et al. (2019) found that the area ratio of the VCN to the FN was significantly correlated with the CAP and IT-MAIS scores at 2 years after CI. In our study, this ratio showed a strong correlation with the CAP and SIR scores, but the correlation was weaker than VCN area. However, due to the presence of thinner FN in some patients, the area ratio of the VCN to the FN was still large despite a thin VCN, which interfered with the accuracy of the results.

Oblique plane sagittal IAC views can show four nerve bundles on MRI: CN, FN, inferior vestibular nerve, and superior vestibular nerve (Govaerts et al., 2003). Since it is difficult to distinguish the CN from other nerves on MRI, Birman et al. (2016) suggested classifying CND according to the number of nerves within the IAC. From oblique plane sagittal IAC views on MRI, IAC nerve grades 0, I, II, and III represent no nerves, one, two, and three nerve bundles, respectively, inside the IAC. These grades correspond to CNA. Grade IV represents four nerves and a thin CN and corresponds to CNH. Previous studies have reported that the IAC nerve grading system was significantly related to the postoperative effect of CI (Birman et al., 2016; Wei et al., 2017; Han et al., 2019). In our study, a higher number of nerve bundles was associated with higher CAP and SIR scores in CND patients. The number of nerve bundles showed strong correlations with the CAP and SIR scores.

In the stability selection analysis for CI outcomes in patients with CND, the VCN area and the number of nerve bundles within the IAC were most representative stable features affecting the CAP and SIR scores at 2 years after CI. For CAP, the accuracy rate was 71% for both the SVM classification and the AUC, whereas for SIR, the accuracy rate for SVM classification and AUC was 93% and 94%, respectively. Only about half of the CND patients in our study were expected to show relative good outcomes (CAP 5-7 or SIR > 1). Our models can help surgeons select the appropriate side for CI and at the same time, provide reasonable expectations regarding the effects of CI surgery. For patients who show inadequate benefit following CI, auditory brainstem implantation should be considered despite the risk of serious complications such as cerebrospinal fluid leakage, meningitis, intracranial bleeding, stroke, cranial nerve damage, and even death (Freeman and Sennaroglu, 2018). In our model, we included only the CND patients with normal cochlea and patients with cochlear malformations and other systemic complications were excluded. Therefore, the application of this model requires complete audiological and imaging evaluations before surgery.

## Conclusion

CI in CND with normal cochlea is associated with variable outcomes. We observed that postoperative CAP and SIR scores of CND patients showed a strong correlation with the VCN area and the number of nerve bundles within the IAC. However, age at implantation and residual hearing did not show any strong correlation. Results from our study can help surgeons select the appropriate side for CI and provide reasonable expectations regarding the outcomes of CI surgery.

## Data Availability Statement

The raw data supporting the conclusions of this article will be made available by the authors, without undue reservation.

## Ethics Statement

The studies involving human participants were reviewed and approved by the Research Ethics Board of Tongren Hospital, Beijing, China. Written informed consent to participate in this study was provided by the participants’ legal guardian/next of kin.

## Author Contributions

SLu designed the experiments and collected the data. JX analyzed the data. SLu and JX drafted the manuscript. All authors edited and revised the manuscript and approved the final version of manuscript.

## Conflict of Interest

The authors declare that the research was conducted in the absence of any commercial or financial relationships that could be construed as a potential conflict of interest.

## Publisher’s Note

All claims expressed in this article are solely those of the authors and do not necessarily represent those of their affiliated organizations, or those of the publisher, the editors and the reviewers. Any product that may be evaluated in this article, or claim that may be made by its manufacturer, is not guaranteed or endorsed by the publisher.

## Footnotes

1
https://scikit-learn.org/stable/modules/feature_selection.html#feature-selection
